# Novel findings from family-based exome sequencing for children with biliary atresia

**DOI:** 10.1038/s41598-021-01148-y

**Published:** 2021-11-08

**Authors:** Kien Trung Tran, Vinh Sy Le, Lan Thi Mai Dao, Huyen Khanh Nguyen, Anh Kieu Mai, Ha Thi Nguyen, Minh Duy Ngo, Quynh Anh Tran, Liem Thanh Nguyen

**Affiliations:** 1 Vinmec Research Institute of Stem Cell and Gene Technology, 458 Minh Khai, Hai Ba Trung District, Hanoi, Vietnam; 2 University of Engineering and Technology, Vietnam National University Hanoi, 144 Xuan Thuy, Cau Giay District, Hanoi, Vietnam; 3 Bioequivalence Center, National Institute of Drug Quality Control, 11/157 Bang B, Hoang Mai District, Hanoi, Vietnam; 4 Vinmec International Hospital, 458 Minh Khai, Hai Ba Trung District, Hanoi, Vietnam; 5 Vietnam National Children’s Hospital, 18/879 La Thanh, Dong Da District, Hanoi, Vietnam

**Keywords:** Molecular biology, Genetics, Medical genetics, Mutation

## Abstract

Biliary atresia (BA) is a progressive inflammation and fibrosis of the biliary tree characterized by the obstruction of bile flow, which results in liver failure, scarring and cirrhosis. This study aimed to explore the elusive aetiology of BA by conducting whole exome sequencing for 41 children with BA and their parents (35 trios, including 1 family with 2 BA-diagnosed children and 5 child-mother cases). We exclusively identified and validated a total of 28 variants (17 X-linked, 6 de novo and 5 homozygous) in 25 candidate genes from our BA cohort. These variants were among the 10% most deleterious and had a low minor allele frequency against the employed databases: Kinh Vietnamese (KHV), GnomAD and 1000 Genome Project. Interestingly, *AMER1*, *INVS* and *OCRL* variants were found in unrelated probands and were first reported in a BA cohort. Liver specimens and blood samples showed identical variants, suggesting that somatic variants were unlikely to occur during morphogenesis. Consistent with earlier attempts, this study implicated genetic heterogeneity and non-Mendelian inheritance of BA.

## Introduction

Biliary atresia is a progressive inflammation and fibrosis of the biliary tree that consequently results in the development of cholestatic liver disease. BA was first described by surgeon John Thomson in 1892^1^ and is among the most fatal diseases, leading to severe complications in infants. The disease occurs in the early stage of neonates and can be treated by hepatic portoenterostomy or Kasai operation^2^. After surgical treatment, however, approximately 50% of affected infants require liver transplantation, while the rest would sustain their own liver up to the age of 5–10 years^3^. A study on Vietnamese BA patients reported that 84% and 71% of Kasai-treated patients survived after 1–2 years, respectively. Additionally, the respective ratios were 52% and 28% for the group without Kasai treatment^4^. It is estimated that after hepatic portoenterostomy operation, 70–80% of patients with BA still require liver transplantation by adulthood due to the progressive development of liver scarring, failure and cirrhosis^5,6^.

Although BA has been extensively studied, its aetiology and pathogenesis remain elusive. Several hypotheses explaining the cause of the disease, including viral infection, autoimmune-mediated bile duct destruction, biliary toxin, and genetic abnormality, have been proposed^7^. Regarding genetic aspects, debate over the Mendelian mechanism of the disease has been raised due to a lack of familial BA and a discordant presentation of BA in the monozygotic twin^8^. Nevertheless, some cases with familial BA have been reported, suggesting that either a recessive autosomal inheritance or a combination of genetic and acquired factors might contribute to the disease’s aetiology^9–12^. In addition, some studies have examined an association between BA and microchimerism, where the genetic trait is maternally transferred from the mother and later contributes to phenotypic heterogeneity and non-Mendelian inheritance^13,14^. More specifically, a heterozygous transition CFC1:c.433G > A in 5 BA patients with polysplenia syndrome implies a genetic predisposition to BA splenic malformation^15^. In a mouse model, inactivation of the hepatocyte nuclear factor 1 beta gene (Hnf1β) causes abnormalities of the gallbladder and intrahepatic bile ducts, resulting in severe jaundice^16^. Observations of an increased incidence of BA in some groups, such as Asian and Polynesian populations, suggest that genetic and environmental factors might cause the disease. Recent genetic studies have revealed a linkage between cholestatic jaundice and genetic predispositions in both nuclear DNA and mitochondrial DNA^17–20^.

The prevalence of BA is 1 in 8000–18,000 live births and varies among countries and groups, with a dominance of females over males^21^. The disease occurs more frequently in Southeast Asia and the Ocean Pacific^22^. It is approximately 1 in 5000 live births in Taiwan compared to 1 in 14,000–20,000 in North America or Western Europe^6,23,24^. To our knowledge, there is no epidemiological study of BA in Vietnamese, as is the prevalence of this fatal disease. The prevalence of BA in Vietnam is estimated to be as high as 1 in 2400 live births as equal to that of the Ocean Pacific regions^22^. Although BA and BA-related liver diseases are often observed in Vietnamese infants and are life-threatening diseases, few studies have been reported thus far^4,25,26^. To date, the Kasai portoenterostomy procedure has been introduced as a routine surgical practice and offers a better opportunity to patients^25^. However, a number of patients still require liver transplantation after the operation or have a low quality of life due to the disease’s complications. Recently, next-generation sequencing (NGS), particularly whole exome sequencing (WES), has been increasingly applied for detecting variants in patients with cholestasis^27^. It appears to be a powerful tool to aid diagnosis and to provide timely and accurate therapeutic treatments. Therefore, we aimed to investigate the genetic pattern of BA by conducting a family-based WES for children with BA in hope of exploring new and characterized causative variants, which would shed light on the aetiology of the deadly disease.

## Materials and methods

### Patient recruitment

BA diagnosis was based on intraoperative findings and liver biopsy. Patients with confirmed BA and their parents were recruited at Vinmec International Hospital and Vietnam National Hospital of Pediatrics in Hanoi from May 2019 to May 2020. A written informed consent form was provided to the parents for their participation. The study was approved by the Ethics Committee of the hospitals in accordance with the Declaration of Helsinki.

### Sample collection and DNA extraction

Approximately 2 mL of peripheral blood from patients and their parents was collected in an EDTA anticoagulant tube and stored at − 80 °C. Liver wedge specimens were collected from the Kasai operation, snapped frozen in liquid nitrogen and stored at − 80 °C. Genomic DNA was extracted by using a DNA Mini Blood Isolation Kit based on the manufacturer’s protocol (Qiagen, Germany). DNA samples were quantified by fluorescence using a Qubit BR Quantification Kit (Invitrogen, USA). Extracted DNA samples were preserved at − 80 °C for future uses.

### Whole exome sequencing

Exome sequencing libraries were prepared by using a Nextera Rapid Capture Kit (Illumina, Calif, USA) based on the manufacturers’ protocol with slight modifications. The library concentration was quantified by a Qubit dsDNA Broad Range Assay Kit (Invitrogen, USA). Library size was measured by using a LabChip 3 K Hisense Kit (PerkinElmer, USA). Paired-end exome sequencing with 150 bp cycles was performed on a HiSeq 4000 (Illumina, Calif, USA), targeting an averaged depth of 100X.

### Bioinformatics analysis

Variant calling and annotation were performed based on highly regarded tools^28^. Reads with low quality, adapters and noise were removed prior to the downstream analysis by using FastQC and Trimmomatic. Reads were aligned with the reference genome GRch38 version^29^. Bowtie2, BWA and Qualimap were used for quality control^30^. To minimize the false-positive rate, multiple variant calling tools, including the Genome Analysis Toolkit (GATK)^31^, SAMtools mpileup^32^ and Freebayes^33^, were mutually used.

### Variant classification, functional prediction and genotype–phenotype analysis

A stringent strategy was applied for variant classification, including (i) inclusion of rare and nonsynonymous variants with a minor allele frequency (MAF) < 1% against three databases: the Kinh Vietnamese population (KHV) obtained from our previous study on the Vietnamese human genome database^34^, GnomAD (https://gnomad.broadinstitute.org/) and 1000 Genome Project^29^; (ii) inclusion of variants with 3 types of inheritance modes: X-linked, homozygous and putative de novo; and (iii) variants with a CADD Phred score of > 10, indicating the 10% most deleterious variants in the genome^35^. In silico prediction tools, including SIFT^36^, PolyPhen-2^37^, Mutation Taster^38^, I-Mutant^39^ and HOPE^40^, were employed to predict the impact of genetic changes. Molecular Signatures Databases (MSigDB v7.2) were used to compute the candidate genes with the gene sets of human phenotype ontology^41,42^.

### Validation of WES results

Selected variants were then confirmed by bidirectional Sanger sequencing. Proper primers were designed for these variants, followed by PCR amplification and sequencing on an ABI 3500 DX system using a BigDye Terminator v3.1 (Thermo Fisher, Massachusetts, USA).

### Ethics approval

The study was approved by the Ethics Committee of Vinmec International General Hospital JSC (Decision No. 48/2019/QD-VMEC).

### Consent to participate

A written informed consent form was provided to the parents prior to their participation.

### Consent for publication

The participants provided consent for publication of all relevant data and this manuscript.

## Results

### Clinical features

We recruited a total of 42 children who had been diagnosed with BA based on intraoperative findings and liver biopsy. All patients showed typical BA symptoms, such as prolonged jaundice, acholic stool and abnormalities of the biliary tract at early infantile. The patients, including 23 males and 19 females born from 2009 to 2019, but the majority of patients were born in recent years. All patients underwent Kasai surgery immediately after birth (mostly after their 2 months of life), but the concentrations of bilirubin and serum enzymes indicating liver function, such as ALP, ALT, AST and γ-GT, remained high at the time of enrolment (Table 1). Some patients have developed liver cirrhosis (BA002_3, BA005, BA012, BA013 and BA018). One BA patient has infected with CMV (BA037). Several probands whose siblings were reported to develop liver diseases or other genetic conditions, including BA (BA002_4), primary sclerosing cholangitis (BA032), choledochal cyst (BA042) and haemophilia (BA025). Four mothers experienced abnormal pregnancy (BA024, BA027; BA036, BA038). The remaining families did not show any significant concern during their pregnancy and had no family history of BA or other genetic conditions. Excluding one family who failed to come for blood drawing after the first health examination, we were finally able to collect blood samples from 41 BA-affected children and their parents. Among these 41 children, we collected liver specimens from 18 children obtained from the Kasai operation.

**Table 1:** Clinical features of children with biliary atresia.

Proband	Birth year	Sex	Age diagnosed	Blood test (at the time of enrolment)							Family history, clinical description
				ALP (124–341 IU/L)	ALB (36–50 g/L)	ALT (< 50 IU/L)	AST (< 50 IU/L)	γ-GT (12–123 IU/L)	T-Bil (2–20 µmol/L)	D-Bil (< 8.6 µmol/L)	
BA001	2016	F	1 m/o	N/A	N/A	190.5	211.9	30.9	73.59	36.8	1st child; No family history of BA or other genetic disease
BA002_3	2014	F	1.5 m/o	N/A	34	57.8	109.9	168.4	143.8	83	Her younger brother was diagnosed with BA; Currently, she developed signs of cirrhosis
BA002_4	2018	M	2 m/o	439	44.8	163.3	205.4	1212.4	289.6	150.8	His sister (BA002_3) showed similar CJ symptoms and diagnosed with BA
BA003	2018	F	50 days	N/A	N/A	259.3	289.9	N/A	210.7	111.5	2nd child; Her grandfather's daughter died at 1 m/o and showed pale stool
BA004	2010	M	2 m/o	677	43.3	282.5	301.5	820	20	5.4	3rd child; No family history of BA or other genetic disease; Splenomegaly; stool with fresh blood
BA005	2011	M	45 days	501	36.8	153.3	166.7	243.3	15.7	4.6	2nd child, full term, born via C-section with birthweight of 3.5 kg; No family history of BA or other genetic disease; Developed cirrhosis after Kasai operation
BA006	2015	M	2 m/o	N/A	37.1	107.3	206.8	200	132.7	73.3	1st child; No family history of BA or other genetic disease
BA007	2017	M	2 m/o	275	41.1	66.1	85.8	58.8	8.8	2.2	1st child; No family history of BA or other genetic disease
BA009	2018	M	1.5 m/o	777.2	30.7	167.6	249.7	410.5	238.1	131.7	3rd child; No family history of BA or other genetic disease
BA010	2010	F	1 m/o	668	40.1	173.4	129.2	249.9	25.7	5.9	1st child, full term, C-section delivered with birthweight of 3.4 kg; No family history of BA or other genetic disease
BA011	2012	M	1 m/o	310	45	50	67.2	66.2	7.9	1.3	2nd child; Vaginal delivered; No family history of BA or other genetic disease
BA012	2015	F	2.5 m/o	748	41.2	876	585.2	624.4	58.2	30.6	2nd child; No family history of BA or other genetic disease. Cirrhosis developed; Splenomegaly
BA013	2010	F	1 m/o	249	40	38.2	52.6	57.3	21.8	4.3	1st child; No family history of BA or other genetic disease; Cirrhosis developed
BA014	2016	F	2 m/o	N/A	42.2	109.5	87.8	201.7	8.9	2.2	1st child; C-section delivered with birthweight of 3.4 kg; No family history of BA or other genetic disease
BA015	2016	F	1 m/o	N/A	39.5	167.8	115.8	460	80.3	46.4	2nd child; No family history of BA or other genetic disease
BA016	2014	M	3 m/o	353.8	37.09	110.4	196.1	424.1	16.8	5.7	1st child; No family history of BA or other genetic disease
BA017	2016	F	2 m/o	516	34.6	161.5	282.7	224.6	56.8	26.8	2nd child; No family history of BA or other genetic disease
BA018	2015	M	2 m/o		33.5	82.9	191.2	371	180.8	107.9	2nd child; No family history of BA or other genetic disease; Prolonged jaundice, acholic stool; cirrhosis after Kasai operation
BA019	2017	F	3 m/o	1195	29.7	64.9	150.9	384.1	38.8	16.9	1st child; No family history of BA or other genetic disease
BA020	2009	M	2 m/o	386	39.8	87.4	80.7	176.7	16.7	5.9	1st child; No family history of BA or other genetic disease
BA021	2018	F	65 days	584.8	37.6	220.9	323.1	918.9	224.1	123	1st child; No family history of BA or other genetic disease
BA023	2018	M	3 m/o	635.7	36.75	163.9	258.5	404	153.9	85	2nd child; No family history of BA or other genetic disease
BA024	2017	F	2 m/o	280.3	33.2	63.9	66.9	88	14.7	5	A child from 2nd pregnancy; C-section delivered, full term; 1st pregnancy was a boy, stillbirth at 5 m/o of gestation due to a low level of amniotic fluid. No family history of BA or other genetic disease
BA025	2018	F	3 m/o	300	41.2	175.8	226.3	465.1	131.1	82.3	3nd child; her older brother was with haemophilia; her older sister was healthy
BA026	2018	M	2 m/o	498	43.2	178.7	240.5	781	76.7	52	1st child; No family history of BA or other genetic disease
BA027	2018	M	40 days	497	38.5	78	104.9	565.1	11.3	4.1	He was a child from his mother’s 3rd pregnancy; the 1st pregnancy was stillbirth at 7 weeks of gestation due to no heartbeat; the 2nd was a molar pregnancy discovered at 8 weeks of gestation
BA028	2016	M	28 days	421	36.5	63.7	80.2	171.6	9.2	2.4	1st child of healthy parents. His paternal grandfather developed liver cirrhosis at age of 50
BA029	2014	M	1 m/o	N/A	39.5	221.6	227.8	527.7	89.9	51.7	1st child; Full term, C-section delivered with birthweight of 3.2 kg; No family history of BA or other genetic disease. Prolonged jaundice, acholic stool
BA030	2018	M	1 m/o	404	31.4	123.6	210.2	900.3	208	120.4	2nd child; No family history of BA or other genetic disease
BA031	2018	M	15 days	556	35.8	56.9	142.6	855.5	143.7	80.3	2nd child; No family history of BA or other genetic disease
BA032	2018	M	1 m/o	427.4	34.4	76.4	144.6	144.6	150.6	69.6	He was the 2nd child. The first child was diagnosed with primary sclerosing cholangitis and died at 28 m/o
BA033	2018	F	2.5 m/o	648	32.8	134.7	255.2	131.8	368.8	188	2nd child; No family history of BA or other genetic disease
BA034	2018	F	29 days		35.2	44.2	150.8	N/A	104.9	60.9	2nd child; No family history of BA or other genetic disease
BA035	2015	F	72 days	374	33.1	82.5	173.3	70	278.8	142	1st child; No family history of BA or other genetic disease
BA036	2018	F	1.5 m/o	311	39.7	164.6	265.9	280.4	131.3	97.8	She was a child from her mother's 2nd pregnancy. The first pregnancy was miscarriage
BA037	2018	M	1 m/o	808.3	38.9	246.7	317.8	329.5	139.8	85.8	1st child; He was infected with CMV. His father was infected with HBV. No family history of BA or other genetic disease
BA038	2018	M	66 days	240	39.8	113.8	87.2	833	81.4	47.3	Full term, vaginal delivered with birthweight of 3.1 kg. He was a child from his mother's 3rd pregnancy; The 1st and 2nd pregnancy were stillbirth at 8 weeks of gestation. He had an inguinal hernia
BA039	2019	F	40 days	618	36.2	115.4	130.7	899.3	130.3	101.2	She was a child of her mother’s 2nd pregnancy; the first was aborted. Her maternal grandfather was with hepatitis
BA040	2019	M	2 m/o	629.2	37.7	93.4	220	604.1	256.7	137	1st child; No family history of BA or other genetic disease
BA041	2019	M	28 days	320	42.6	138.6	265.5	905.2	108	82.7	1st child; No family history of BA or other genetic disease. His prenatal grandmother was infected with HBV
BA042	2019	F	1 m/o	N/A	N/A	211	663	N/A	229	120	2nd child; the first child was a healthy boy. Her mother was diagnosed with choledochal cyst at age of 13

*M* male, *F* female, *m/o* month old, *ALP* alkaline phosphatase, *ALB* albumin, *ALT* alanine aminotransferase, *AST* aspartate aminotransferase, *γ-GT* gamma-glutamyl transferase, *T-Bil* total bilirubin, *D-Bil* direct bilirubin, *HBV* hepatitis B, *CMV* cytomegalovirus, *CJ* cholestatic jaundice, *N/A* not available, BA002_3 and BA002_4 were siblings.

### Genetic properties

**Figure 1:**
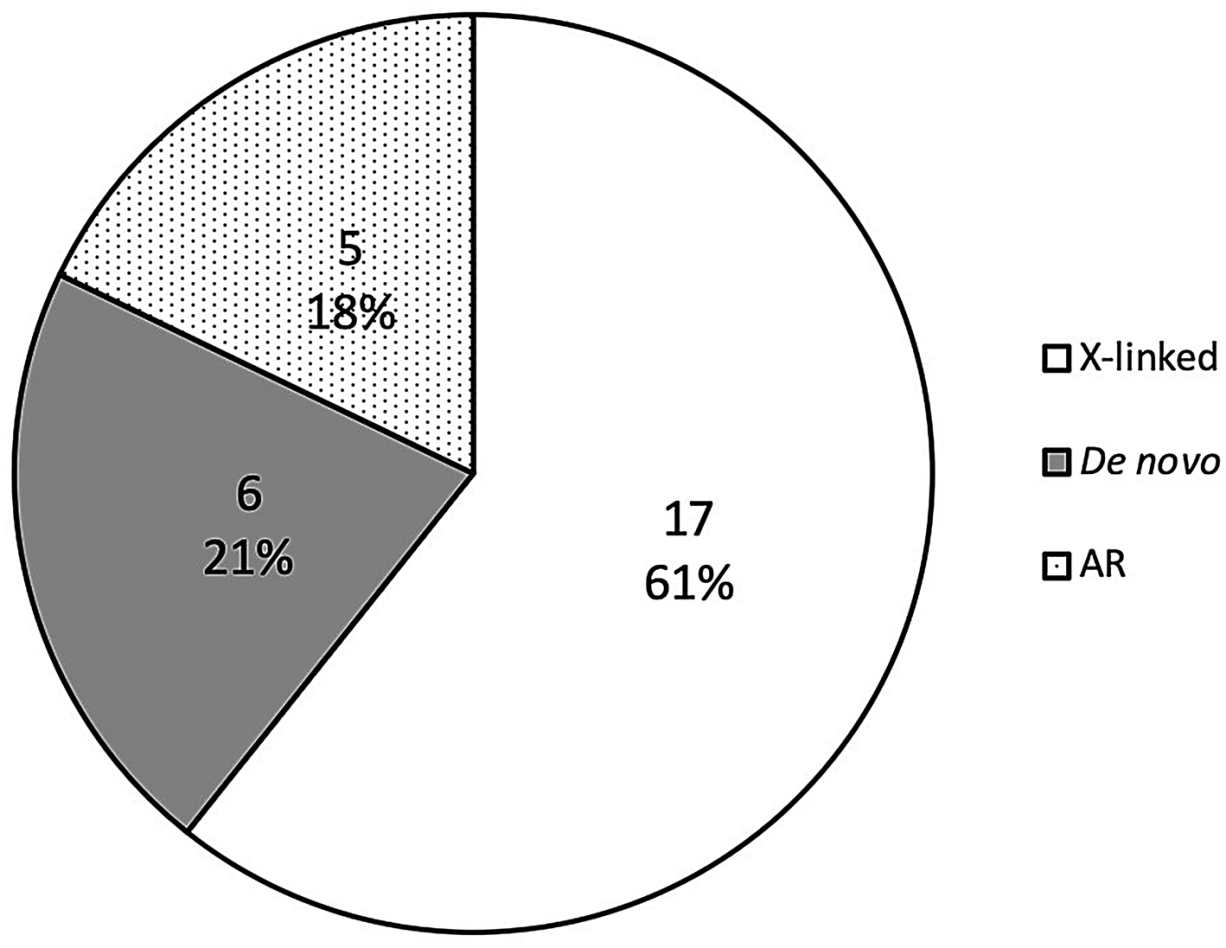
Mode of inheritance of identified variants from the biliary atresia cohort*.* X-linked variants are presented in blank; de novo variants are presented in grey; and autosomal recessive variants are presented in dots.

We applied a strict filtering strategy by removing variants with MAF > 1%, synonymous variants and variants with a CADD scaled score < 10. Finally, we identified a total of 28 variants in 25 genes from our BA-affected cohort (Table 2). All variants were subsequently confirmed by Sanger sequencing (Fig. S1). Among the 28 detected variants, 17 X-linked variants (61%) were detected in 17 different genes, 6 de novo variants (21%) were detected in 6 genes from 5 probands, including *INVS*, *ELP2*, *TINAG*, *CEP63, CCDC136*, and *BCAR1*, and 5 homozygous variants were identified in 5 genes (18%) (Fig. 1), including *HACE1*, *VPS13C*, *RAPGEF4*, *FOCAD* and *INVS* (Table 2). Family #2 involved two siblings with similar phenotypes (early onset jaundice, BA diagnosed). Two X-linked and 1 homozygous variants were detected in the male sib of family #2, and none were detected in his sister (Table 2). Interestingly, several genes with genetic predisposition were observed in unrelated patients, including *AMER1* (BA004 and BA007), *INVS* (BA014 and BA041), and *OCRL* (BA032 and BA041). Noticeably, proband BA014 carried an *INVS *de novo variant, while proband BA041 carried an *INVS* homozygous variant (Table 2).

**Table 2:** Genetic characteristics of Vietnamese children with biliary atresia.

Proband	Sex (M/F)	Chr	Position	Gene	DNA change	MOI	Genotype				MAF (KHV/GnomAD East Asian/1 KG)		CADD (Phred)
							Proband	UM	UF	AS	Allele frequency	#homozygotes	
BA002_4	M	chr6	104,771,988	*HACE1*	NM_001350555:c.G1660 > A	AR	T/T	C/T	C/T	C/T	0.01/0.001731/0.001996	0	26.2
		chrX	72,684,557	*PHKA1*	NM_001122670:c.G478 > A	X-linked	T	C/T	C	C/T	0.008/0.001949/0.005298	0	23.2
		chrX	123,665,767	*THOC2*	NM_001081550:c.G1261 > A	X-linked	T	C/T	C	C/T	0.008/0.01483/0.01589	0	21.6
BA004	M	chrX	123,888,703	*XIAP*	NM_001167:c.C962 > G	X-linked	G	G/C	N/A		0/0.003356/0.003958	0	29.5
BA007	M	chr15	61,929,659	*VPS13C*	NM_017684:c.C5999 > G	AR	C/C	G/C	G/C		0.01/0.005391/0.00998	0	24.3
		chrX	64,192,212	*AMER1*	NM_152424:c.A1075 > T	X-linked	A	T/A	T		0/0.0002802/0	0	25
		chrX	77,508,398	*ATRX*	NM_138270:c.C7318 > G	X-linked	C	G/C	G		0/0.0005613/0.001323	0	22.3
		chrX	85,367,724	*POF1B*	NM_001307940:c.A325 > C	X-linked	G	T/G	T		0.003/0.001392/0.001321	0	19.47
BA009	M	chrX	130,015,441	*BCORL1*	NM_001184772:c.G2669 > A	X-linked	A	G/A	G		0.008/0.001949/0.002635	0	23.3
BA014	F	chr9	100,252,390	*INVS*	NM_001318382:c.C208 > T	De novo	C/T	C/C	N/A		0/0/0	0	37
		chrX	40,073,898	*BCOR*	NM_001123383:c.C1448 > T	X-linked	A/A	G/A	N/A		0.003/0.003068/0.001321	0	23.1
BA016	M	chrX	56,565,305	*UBQLN2*	NM_013444:c.C1432 > G	X-linked	G	C/G	C		0/0/0	0	23.7
BA020	M	chrX	43,693,330	*MAOA*	NM_000240:c.G208 > A	X-linked	A	G/A	G		0/0/0	0	22.6
		chrX	108,733,510	*IRS4*	NM_003604:c.G2835 > C	X-linked	G	C/G	C		0.003/0/0	0	22.8
BA028	M	chr18	36,156,524	*ELP2*	NM_001242879:c.C1124 > T	De novo	C/T	C/C	C/C		0/0/0	0	26.3
BA032	M	chr2	173,016,403	*RAPGEF4*	NM_001282901:c.C1204 > A	AR	A/A	C/A	C/A		0/0.0001925/0	0	23.2
		chrX	129,590,191	*OCRL*	NM_001587:c.T2603 > A	X-linked	A	T/A	T		0.003/0/0	0	18.03
BA033	F	chr6	54,308,777	*TINAG*	NM_014464:c.C227 > T	De novo	C/T	C/C	C		0/0/0	0	22.2
BA035	F	chr3	134,561,515	*CEP63*	NM_001042383:c.C1468 > A	De novo	C/A	C/C	C/C		0/0/0	0	23.5
		chr7	128,812,751	*CCDC136*	NM_022742:c.C2585 > A	De novo	C/A	C/C	C/C		0/0/0	0	14.62
BA036	F	chr16	75,243,074	*BCAR1*	NM_001170715:c.C83 > T	De novo	G/A	G/G	G/G		0/0/0	0	29.4
BA037	M	chr9	20,948,857	*FOCAD*	NM_017794:c.C3805 > A	AR	A/A	C/A	C/A		0.005/0.0005763/0.001996	0	23.2
		chrX	70,341,839	*KIF4A*	NM_012310:c.A1174 > C	X-linked	C	A/C	A		0/0.005278/0.001379	0	25.1
BA038	M	chrX	47,448,875	*ZNF41*	NM_001324139:c.C637 > T	X-linked	A	G/A	G		0.003/0/0	0	15.37
BA040	M	chrX	3,112,294	*ARSF*	NM_001201538:c.C1511 > T	X-linked	T	C/T	C		0.003/0/0	0	22.8
		chrX	64,191,164	*AMER1*	NM_152424:c.C2123 > A	X-linked	T	G/T	G		0.003/0/0	0	14.16
BA041	M	chr9	100,126,394	*INVS*	NM_014425:c.C118 > G	AR	G/G	C/G	C/G		0.007/0.004996/0.005988	0	22.7
		chrX	129,557,351	*OCRL*	NM_000276:c.G265 > C	X-linked	C/C	G/C	G		0.007/0.0008396/0.002639	0	24.1

*Chr* chromosome, *M* male, *F* female, *A.A* amino acid, *MOI* mode of inheritance, *UM* unaffected mother, *UF* unaffected father, *AS* affected sibling, *N/A* not available, *MAF* minor allele frequency, *KHV* Kinh Vietnamese, *1 KG* 1000 Genome Project, *CADD* scaled score.

In addition to blood samples, we were able to collect 18 liver specimens from our BA cohort. Of these, blood and liver samples from 8 children shared identical variants (BA009, BA016, BA032, BA036, BA037, BA038, BA040 and BA041). Additionally, we did not detect any significant variants based on our rationales for variant classification (Table 3). In other words, this study did not detect any somatic variants from liver samples.

**Table 3:** Identical variants detected from blood and liver samples.

Proband	Sex (M/F)	Chr	Position	SNP ID	Ref	Alt	Gene	DNA change	A.A change	MOI	Genotype		
											Proband	UM	UF
BA003	F							Undetected					
BA005	M							Undetected					
BA009	M	X	130,015,441	rs201843717	G	A	*BCORL1*	NM_001184772:c.G2669 > A	p.Arg325Gln	X-linked	A	G/A	G
BA016	M	X	56,565,305		C	G	*UBQLN2*	NM_013444:c.C1432 > G	p.Pro478Ala	X-linked	G	C/G	C
BA021	F							Undetected					
BA023	M							Undetected					
BA025	F							Undetected					
BA030	M							Undetected					
BA031	M							Undetected					
BA032	M	2	173,016,403	rs773527960	C	A	*RAPGEF4*	NM_001282901:c.C1204 > A	p.Gln622Lys	AR	A/A	C/A	C/A
		X	129,590,191	rs752439587	T	A	*OCRL*	NM_001587:c.T2603 > A	p.Met876Lys	X-linked	A	T/A	T
BA035	F							Undetected					
BA036	F	16	75,243,074	rs1327850193	G	A	*BCAR1*	NM_001170715:c.C83 > T	p.Ala10Val	De novo	G/A	G/G	G/G
BA037	M	9	20,948,857	rs544335294	C	A	*FOCAD*	NM_017794:c.C3805 > A	p.Pro1269Thr	AR	A/A	C/A	C/A
		X	70,341,839	rs371383515	A	C	*KIF4A*	NM_012310:c.A1174 > C	p.Asn392His	X-linked	C	A/C	A
BA038	M	X	47,448,875	rs758443040	G	A	*ZNF41*	NM_001324139:c.C637 > T	p.Arg299Cys	X-linked	A	G/A	G
BA039	F							Undetected					
BA040	M	X	3,112,294	rs1426850924	C	T	*ARSF*	NM_001201538:c.C1511 > T	p.Pro504Leu	X-linked	T	C/T	C
		X	64,191,164	rs764261510	G	T	*AMER1*	NM_152424:c.C2123 > A	p.Thr708Asn	X-linked	T	G/T	G
BA041	M	9	100,126,394	rs148219510	C	G	*INVS*	NM_014425:c.C118 > G	p.Leu40Val	AR	G/G	C/G	C/G
		X	129,557,351	rs753369725	G	C	*OCRL*	NM_000276:c.G265 > C	p.Asp89His	X-linked	C/C	G/C	G

*Chr* chromosome, *M* male, *F* female, *Ref* reference, *Alt* alternative, *A.A* amino acid, *MOI* mode of inheritance, *AR* autosomal recessive, *UM* unaffected mother, *UF* unaffected father.

### Effect of genetic predisposition

The detected variants showed extremely low MAFs against three employed databases: Kinh Vietnamese (KHV), GnomAD and 1000 Genome Project (Table 2). We noticed that the MAFs of the HACE1 and VPS13C variants were above 1% against the KHV database, while the rest were significantly below the thread hold of 1%. All variants with CADD Phred scaled scores were above 10 and mostly above 20, indicating either the 10% or 1% most deleterious substitutions, respectively. Among these variants, INVS:c.C208 > T was the most deleterious, with the highest scaled score of 37 (Table 2).

The Polyphen-2 and SIFT tools showed a consensus on the damaging impact *of HACE1, PHKA1, XIAP,* and *AMER1* (c.A1075 > T), *POF1B, MAOA, BCAR1, FOCAD, ARSF* and *OCRL* variant, while the rest varied from tools (Table S1). We used I-Mutant to predict the stability of amino acid substitution for 28 identified variants via the change of free energy change values (DDG). The results show that except OCRL:c.T2603 > A(p.Met876Lys), which increased the stability of the mutant compared to that of the wild-type variants, all variants showed decreased stability (Table S2). The HOPE tool was used to predict the structural effect of missense variants, showing changes in residue size and hydrophobic and structural stability (Fig. S2). Changes in amino acid size and charge resulted in a loss of interaction and disturbance of protein function. Several variants, for example HACE1:c.G1660 > A(pAla651Thr) and PHKA1:c.G478 > A(p.Asp160Asn), whose wild-type residues are located in important domains. Thus, any substitution in these regions was predicted to lead to a functional disturbance. In contrast to I-mutant prediction, HOPE showed that an alternation of methionine by lysine residue in the variant OCRL:c.T2603 > A(p.Met876Lys) can disturb the hydrophobic interaction of the altered residue with other molecules on the surface of the protein (Fig. S2).

### Analysis of biological function and human disease phenotype

Compute overlaps of 25 candidate genes to the human phenotype ontology from the Molecular Signatures Database, involving 4,494 gene sets (FDR q value < 0.05), indicated that the candidate genes felt into various human phenotype gene sets, ranging from gonosomal inheritance and X-linked recessive inheritance to involuntary movements (Table 4). We also computed our gene set to find the association of these genes with the reported phenotypes available from the HPO and Monarch Initiative (Table S3). However, we did not find any overlapping phenotypes from these databases. The reason might be a lack of genes/pathways associated with the BA phenotype in these available databases, which are often dominated by studies on Caucasians, where the prevalence of BA in this group is much lower than that in Asians. By applying the same strategy to identify the potential contribution of ciliary dysgenesis underlying the BA phenotype, we used a gene set containing 2016 genes of interest^43^. We found that some genes from our study, including *BCOR*, *INVS* and *OCRL,* were included in this gene set. This result suggested the novelty of *BCOR*, *INVS* and *OCRL* from our BA cohort.

**Table 4:** Analysis of human phenotype ontology.

Gene set name	# genes in gene set (K)	Description	# genes in overlap (k)	k/K	*p* value	FDR q-value	Gene overlap
HP_GONOSOMAL_INHERITANCE	253	Gonosomal inheritance	12	0.0474	1.49E−20	6.70E−17	*ATRX, OCRL, THOC2, MAOA, KIF4A, BCORL1, XIAP, POF1B, PHKA1, BCOR, AMER1, UBQLN2*
HP_X_LINKED_RECESSIVE_INHERITANCE	173	X-linked recessive inheritance	9	0.052	8.13 E−16	1.83 E−12	*ATRX, OCRL, THOC2, MAOA, KIF4A, BCORL1, XIAP, POF1B, PHKA1*
HP_SELF_INJURIOUS_BEHAVIOR	108	Self-injurious behaviour	6	0.0556	5.66 E−11	8.47 E−08	*ATRX, OCRL, THOC2, MAOA, BCOR, ELP2*
HP_ABNORMAL_EMOTION_AFFECT_BEHAVIOR	415	Abnormal emotion/affect behaviour	7	0.0169	4.97 E−09	4.97 E−06	*ATRX, OCRL, THOC2, MAOA, BCOR, ELP2, VPS13C*
HP_NEUROLOGICAL_SPEECH_IMPAIRMENT	1022	Neurological speech impairment	9	0.0088	6.24 E−09	4.97 E−06	*ATRX, OCRL, KIF4A, BCOR, AMER1, UBQLN2, ELP2, ZNF41HACE1*
HP_DELAYED_SPEECH_AND_LANGUAGE_DEVELOPMENT	696	Delayed speech and language development	8	0.0115	6.63 E−09	4.97 E−06	*ATRX, THOC2, BCORL1, AMER1, ELP2, ZNF41, HACE1, CEP63*
HP_AUTISTIC_BEHAVIOR	450	Autistic behaviour	7	0.0156	8.68 E−09	5.12 E−06	*ATRX, THOC2, MAOA, BCORL1, BCOR, VPS13C, ZNF41*
HP_ABNORMAL_AGGRESSIVE_IMPULSIVE_OR_VIOLENT_BEHAVIOR	251	Abnormal aggressive, impulsive or violent behaviour	6	0.0239	9.12 E−09	5.12 E−06	*ATRX, OCRL, THOC2, MAOA, BCOR, ELP2*
HP_SHORT_STATURE	1152	Short stature	9	0.0078	1.75 E−08	8.76 E−06	*ATRX, OCRL, THOC2, BCOR, AMER1, ELP2, HACE1, CEP63, INVS*
HP_INVOLUNTARY_MOVEMENTS	905	Involuntary movements	8	0.0088	5.05 E−08	2.19 E−05	*ATRX, OCRL, THOC2, MAOA, BCORL1, UBQLN2, ELP2, VPS13C*

Similar to a previous study^43^, we did not identify any variants in some genes that have been previously suggested to be associated with BA or BA-related diseases, such as *PKD2* (polycystic kidney disease 2, polycystic kidney and hepatic disease 1), *CFC1* (polysplenia), *JAG1* (Alagille syndrome) and *PKD1L1* (biliary atresia splenic malformation syndrome- BASM). We also did not find significant variants in the susceptibility loci of *ADD3*, *XPNPEP1*, *GPC1*, *ARF6* and *EFEMP1,* as suggested by GWAS^44^.

## Discussion

Similar to other previous studies, we attempted to reveal the genetic pattern of BA disorder by conducting trio-based exome sequencing for 40 families involving 41 children with BA. Going beyond this establishment in a genetic study for such a rare and complex disorder, we further tested our hypothesis of whether the detected variants occurred in somatic or germline cells by sequencing both blood and available liver specimens obtained from our BA cohort. Due to the complexity of BA, we applied a stringent bioinformatics pipeline and tight quality control to determine either the rarest variants or putative de novo events from our BA cohort, which would avoid a huge number of variants as often experienced from mass sequencing. Taking this straightforward principle enabled us to end up with a total of 28 variants in 25 respective genes. Identical variants detected from blood and liver samples allowed us to rule out the occurrence of somatic variants in the development of the disease as previously hypothesized^45^.

In agreement with previous studies, our results showed an intriguing genetic aspect of BA, which was highly heterogeneous. It is worth noting that along with other variants, this study found 3 genes whose variants occurred in unrelated probands, including *AMER1*, *INVS* and *OCRL*. While the aetiology of BA remained unclear and was unlikely to follow the Mendelian model, our results implicated their role in the disease's development. Overlapping findings of *BCOR*, *INVS* and *OCRL* in the Vietnamese BA cohort with a large comprehensive ciliopathy and biliary genes of interest in the previous study^43^ further supported the possibility of the causative role of these genes in BA. *AMER1* (MIM#300647) encodes APC membrane recruitment protein 1, which acts as an inhibitor of the canonical Wnt/beta-catenin signalling pathway^46^ and controls hepatobiliary development during embryogenesis. In mature healthy liver cells, it is mostly inactive, and the abnormal Wnt/beta-catenin signalling pathway can promote the development of liver diseases^47^. *AMER1* associates with osteopathia striata with cranial sclerosis^48^ and Wilms tumour development^49–51^. The gene is involved in the activation of the Wnt/beta-catenin signalling pathway, which drives hepatocarcinoma and cholangiocarcinoma^52^. In addition, analysis of the effect of genetic predispositions of *AMER1* variants indicated that they were damaging because the alternated residues were located in highly conserved positions. The alternations might lead to destabilization of the local conformation and a loss of protein interaction (Table S1, S2, Fig. S2). Despite a lack of *AMER1* to typical BA phenotypes, we inferred its indirect role in the development of BA as a result of activation of the Wnt/beta-catenin signalling pathway.

Our study highly suggested *INVS* as a BA candidate gene owing to *INVS* variant detection in 2 unrelated probands, their mode of inheritance and the effect of genetic predisposition. In particular, INVS: c.C208 > T (p.Arg396*) was de novo*,* and a loss-of-function variant with a CADD score of 37 and its allele frequency was absent from all employed databases. *INVS* encodes inversin protein, which plays a role in primary cilia function and is involved in the cell cycle. Intriguingly, inactivation of *INVS* in a mouse model shows a significant increase in bilirubin levels compared to that of the wild-type and pathogenic changes in ductal plate malformation in the intrahepatic biliary of the mutant mouse^53^. The association of *INVS* with BA had not been previously established due to an absence of *INVS* variants detected in BA patients^54,55^. However, *INVS* is associated with infantile nephronophthisis type 2^56–58^. In our study, we detected an *INVS* heterozygous de novo variant and a homozygous variant from 2 BA unrelated patients (BA014 and BA041). To our knowledge, this novelty is first reported in BA patients, although future studies are needed to clearly explore the role of *INVS* in BA development. Similar to *BCOR* and *INVS*, *OCRL* encodes inositol polyphosphate 5-phosphatase, which might be involved in primary cilia assembly. *OCRL* has been widely reported to be linked to Lowe and Dent syndrome, where clinical manifestations often overlap with Zellweger spectrum disorders, characterized by low muscle tone, feeding difficulty, seizures and liver dysfunction^59–61^. Likewise, a lack of an association of *OCRL* and BA or liver diseases remains a gap for future investigation.

As a result of a rapidly declining cost of DNA sequencing, dozens of rare and previously undiagnosed genetic disorders are currently detectable. For the last 10 years, NGS technology has revolutionized our understanding of human genetics with a high level of accuracy, cost effectiveness and high throughput capability. NGS is steadily becoming a standard in routine diagnostic practices^62^. In BA studies, mitochondrial DNA has been found to associate with BA, suggesting the role of mitochondria in underling the pathogenic mechanism^17^. WES has revealed dozen candidate genes either encode ATP-binding cassette transporters (the ABC superfamily)^18,19^ or are involved in the Notch signalling pathway, such as *JAG1*^19,63^ and *NOTHC2*^20^. GWAS have highlighted a strong association between BA and some variants in the *ADD3* gene located on 10q24.2^64^. Another subsequent study on 171 BA patients and 1,630 controls of European descent found the strongest signal at rs7099604 in the *ADD3* gene^65^. A significant association was found between variant rs1709535*5* on the *XPNPEP1* gene and the disease^66^. Taken together, the aetiology of BA remains challenging due to the involvement of multiple genes and complex mechanisms. Being encouraged by the pioneers, we provided a concrete genetic aspect obtained from an exome trio-based study of a Vietnamese BA cohort. The findings add to our knowledge of the genetic heterogeneity and complexity of BA disorder.

## Conclusion

The aetiology of BA remains challenging because there is a lack of conclusive evidence despite extensive research and medical practices for hundreds of years. However, the recent development of NGS technology and its application in studies of BA and liver diseases have gradually revealed the hidden genetic picture of BA aetiology, where dozens of BA-associated genes have been found. Our study identified 28 variants in 25 genes (all validated) from 41 children with BA. These variants were in the 10% most deleterious and were either rare or extremely rare in the population genome database. A combination of functional prediction and analysis of biological processes enabled us to suggest these candidate genes for the development of BA, particularly with those detected in unrelated BA individuals, including *AMER1*, *INVS* and *OCRL*. Identical variants detected from blood and liver wedge specimens from each BA individual suggested that somatic variants in the liver cells were unlikely to occur during morphogenesis. Taken together, we highlighted the genetic heterogeneity of BA and ruled out the Mendelian model. Future studies are needed to further explore the roles of these genes in the development of BA.

## Supplementary Information

Supplementary Information.

## Data Availability

Data are available from this manuscript and supplementary information.
