# Supplementary material for: Novel findings from family-based exome sequencing for children with biliary atresia
Source: Sci Rep. 2021 Nov 8;11:21815. doi: 10.1038/s41598-021-01148-y (PMC8575792; doi:10.1038/s41598-021-01148-y)
Supplement: Supplementary file 1 — Supplementary Information. [file 41598_2021_1148_MOESM1_ESM.pdf]

Supplementary Information

Novel findings from family-based exome sequencing for children with biliary atresia

Kien Trung Tran[1*], Vinh Sy Le[1,2*], Lan Thi Mai Dao[1], Huyen Khanh Nguyen[3], Anh Kieu Mai[4], Ha Thi Nguyen[4], Minh Duy Ngo[4], Quynh Anh Tran[5], Liem Thanh Nguyen[1]

[1]Vinmec Research Institute of Stem Cell and Gene Technology, 458 Minh Khai, Hai Ba Trung District, Hanoi, Vietnam.
[2] University of Engineering and Technology, Vietnam National University Hanoi, 144 Xuan Thuy, Cau Giay District, Hanoi, Vietnam.
[3]Bioequivalence Center, National Institute of Drug Quality Control, 11/157 Bang B, Hoang Mai District, Hanoi, Vietnam.
[4]Vinmec International Hospital, 458 Minh Khai, Hai Ba Trung District, Hanoi, Vietnam.
[5]Vietnam National Children's Hospital, 18/879 La Thanh, Dong Da District, Hanoi, Vietnam.

Correspondence: Kien Trung Tran, email: trantrungkien80@gmail.com; Liem Thanh Nguyen, email: v.liemnt@vinmec.com

Table S1. Variant characterization and functional prediction.

**Gene**	**DNA change**	**A.A change**	**Intolerance**	***In silico* prediction**		
			pLI/Z score	SIFT	Polyphen_2	Mutation Taster
HACE1	NM_001350555:c.G1660>A	p.Ala651Thr	0/3.59	D:0.001	D:0.96	D:0.810
PHKA1	NM_001122670:c.G478>A	p.Asp160Asn	0/1.88	D:0.012	D:0.999	D:0.810
THOC2	NM_001081550:c.G1261>A	p.Ala421Thr	1/5.53	T:0.176	B:0.002	D:0.548
XIAP	NM_001167:c.C962>G	p.Ala321Gly	0.92/1.49	D:0.0	D:0.99	D:0.810
VPS13C	NM_017684:c.C5999>G	p.Ala2043Gly	0/-1.29	D:0.0	B:0.283	D:0.537
AMER1	NM_152424:c.A1075>T	p.Ser359Cys	0.85/-0.57	D:0.002	D:0.995	D:0.380
ATRX	NM_138270:c.C7318>G	p.Pro2478Ala	1/3.1	D:0.005	B:0.164	N:0.293
POF1B	NM_001307940:c.A325>C	p.Ser109Arg	0/0.78	D:0.013	B:0.12	N:0.231
BCORL1	NM_001184772:c.G2669>A	p.Arg890Gln	1/2.06	D:0.022	D:0.99	N:0.236
INVS	NM_001318382:c.C208>T	p.Arg396*	0/1.07	.:.	.:.	A:0.810
BCOR	NM_001123383:c.C1448>T	p.Pro483Leu	1/1.88	T:1.0	P:0.861	D:0.442
UBQLN2	NM_013444:c.C1432>G	p.Pro478Ala	0.85/1.5	D:0.021	P:0.519	D:0.504
MAOA	NM_000240:c.G208>A	p.V70M	1/2.38	D:0.002	D:0.921	D:0.810
IRS4	NM_003604:c.G2835>C	p.Trp945Cys	0.58/0.09	D:0.004	B:0.43	D:0.466
ELP2	NM_001242879:c.C1124>T	p.Ala445Val	0/0.58	D:0.004	P:0.493	D:0.810
RAPGEF4	NM_001282901:c.C1204>A	p.Gln622Lys	0.99/1.83	T:0.153	B:0.098	D:0.588
OCRL	NM_001587:c.T2603>A	p.Met876Lys	1/2.96	T:0.448	B:0.001	D:0.346
TINAG	NM_014464:c.C227>T	p.Ala76Val	0/-1.17	T:0.154	B:0.074	D:0.810
CEP63	NM_001042383:c.C1468>A	p.Gln490Lys	0/-0.1	T:0.704	P:0.879	D:0.810
CCDC136	NM_022742:c.C2585>A	p.Ala862Glu	0/1.13	D:0.002	P:0.756	D:0.810
BCAR1	NM_001170715:c.C83>T	p.Ala10Val	1/-0.59	D:0.0	D:0.999	D:0.810
FOCAD	NM_017794:c.C3805>A	p.Pro1269Thr	0/-2.93	D:0.003	D:0.989	D:0.537
KIF4A	NM_012310:c.A1174>C	p.Asn392His	1/2.56	T:0.073	D:0.999	D:0.588
ZNF41	NM_001324139:c.C637>T	p.Arg299Cys	0.04/1.3	D:0.042	B:0.01	N:0.090
ARSF	NM_001201538:c.C1511>T	p.Pro504Leu	0/-0.39	D:0.001	D:0.968	D:0.810
AMER1	NM_152424:c.C2123>A	p.Thr708Asn	0.85/-0.57	D:0.032	B:0.002	N:0.252
INVS	NM_014425:c.C118>G	p.Leu40Val	0/1.07	T:0.204	P:0.56	D:0.810
OCRL	NM_000276:c.G265>C	p.Asp89His	1/2.96	D:0.003	D:0.936	D:0.407

Abbreviations: A.A: amino acid; SIFT (T: tolerated; D: damaging; score value <0.05 is likely damaging/deleterious); Polyphen2 (B: benign; P: possibly damaging; D: damaging; core value close to 1 indicates likely damaging/deleterious); Mutation Taster (A: disease causing automatic; D: polymorphism, probably harmless; N: polymorphism, known to be harmless; score value close to 1 shows a high security of the prediction).

Table S2. Prediction of structural changes.

Gene	A.A change	I-mutant	
		Stability	DDG (kcal/mol)
HACE1	p.Ala651Thr	Decrease	-1.15
PHKA1	p.Asp160Asn	Decrease	-1.71
THOC2	p.Ala421Thr	Decrease	-1.28
XIAP	p.Ala321Gly	Decrease	-0.3
VPS13C	p.Ala2043Gly	Decrease	-1.71
AMER1	p.Ser359Cys	Decrease	-1.6
ATRX	p.Pro2478Ala	Decrease	0.52
POF1B	p.Ser109Arg	Decrease	-0.73
BCORL1	p.Arg890Gln	Decrease	-0.68
INVS	p.Arg396*	N/A	
BCOR	p.Pro483Leu	Decrease	-1.36
UBQLN2	p.Pro478Ala	Decrease	-1.73
MAOA	p.V70M	Decrease	-2.58
IRS4	p.Trp945Cys	Decrease	-0.93
ELP2	p.Ala445Val	Decrease	-1.1
RAPGEF4	p.Gln622Lys	Decrease	-0.35
OCRL	p.Met876Lys	Increase	0.48
TINAG	p.Ala76Val	Decrease	-0.27
CEP63	p.Gln490Lys	Decrease	-0.45
CCDC136	p.Ala862Glu	Decrease	-0.75
BCAR1	p.Ala10Val	Decrease	0.48
FOCAD	p.Pro1269Thr	Decrease	-1.33
KIF4A	p.Asn392His	Decrease	-1.76
ZNF41	p.Arg299Cys	Decrease	-1.08
ARSF	p.Pro504Leu	Decrease	-1.39
AMER1	p.Thr708Asn	Decrease	-0.77
INVS	p.Leu40Val	Decrease	-1.58
OCRL	p.Asp89His	Decrease	-1.94

A.A: amino acid; DDG: free energy change; N/A: not available.

Table S3. Gene function and human phenotype diseases.

Gene	HGNC	Encoding protein	Disease causal (HPO/Monarch Initiative)
AMER1	26837	APC membrane recruitment protein 1	Osteopathia striata with cranial sclerosis
ARSF	721	Arylsulfatase F	NA
ATRX	886	ATRX chromatin remodeler	X-linked ID, MR, alpha-thalassemia, etc.
BCAR1	971	BCAR1 scaffold protein, Cas family member	Exocrine pancreatic carcinoma; Alcoholic pancreatitis
BCOR	20893	BCL6 corepressor	Microphthalmia; Oculofaciocardiodental syndrome; Acute Promyelocytic Leukemia
BCORL1	25657	BCL6 corepressor like 1	Shukla-Vernon syndrome; Non-specific Syndromic ID
CCDC136	22225	Coiled-coil domain containing 136	Dyslexia
CEP63	25815	Centrosomal protein 63	Autosomal Recessive Primary Microcephaly; Seckel Syndrome 6
ELP2	18248	Elongator acetyltransferase complex subunit 2	ID
FOCAD	23377	Focadhesin	Type 2 diabetes mellitus; Cleft lip
HACE1	21033	HECT domain and ankyrin repeat-containing ubiquitin ligase	Spastic Paraplegia
INVS	17870	Inversin	Senior-Loken syndrome, Nephronophthisis 2
IRS4	6128	Insulin receptor substrate 4	Hypothyroidism; Congenital, nongoitrous
KIF4A	13339	Kinesin family member 4A	Non-syndromic X-linked ID;
MAOA	6833	Monoamine oxidase A	Brunner syndrome; Monoamine Oxidase A Deficiency
OCRL	8108	OCRL inositol polyphosphate-5-phosphatase	Oculocerebrorenal syndrome; Dent disease type 2
PHKA1	8925	Phosphorylase kinase	Glycogen storage disease
POF1B	13711	POF1B actin binding protein	Primary ovarian failure
RAPGEF4	16626	Rap guanine nucleotide exchange factor 4	Schizophrenia; Moyamoya disease; Heart failure
THOC2	19073	THO complex 2	X-linked ID-short stature-overweight syndrome
TINAG	14599	Tubulointerstitial nephritis antigen	NA
UBQLN2	12509	Ubiquilin 2	Amyotrophic Lateral Sclerosis
VPS13C	23594	Vacuolar protein sorting 13 homolog C	Parkinson Disease
XIAP	592	X-linked inhibitor of apoptosis	Lymphoproliferative Syndrome
ZNF41	13107	Zinc finger protein 41	Non-syndromic X-linked ID

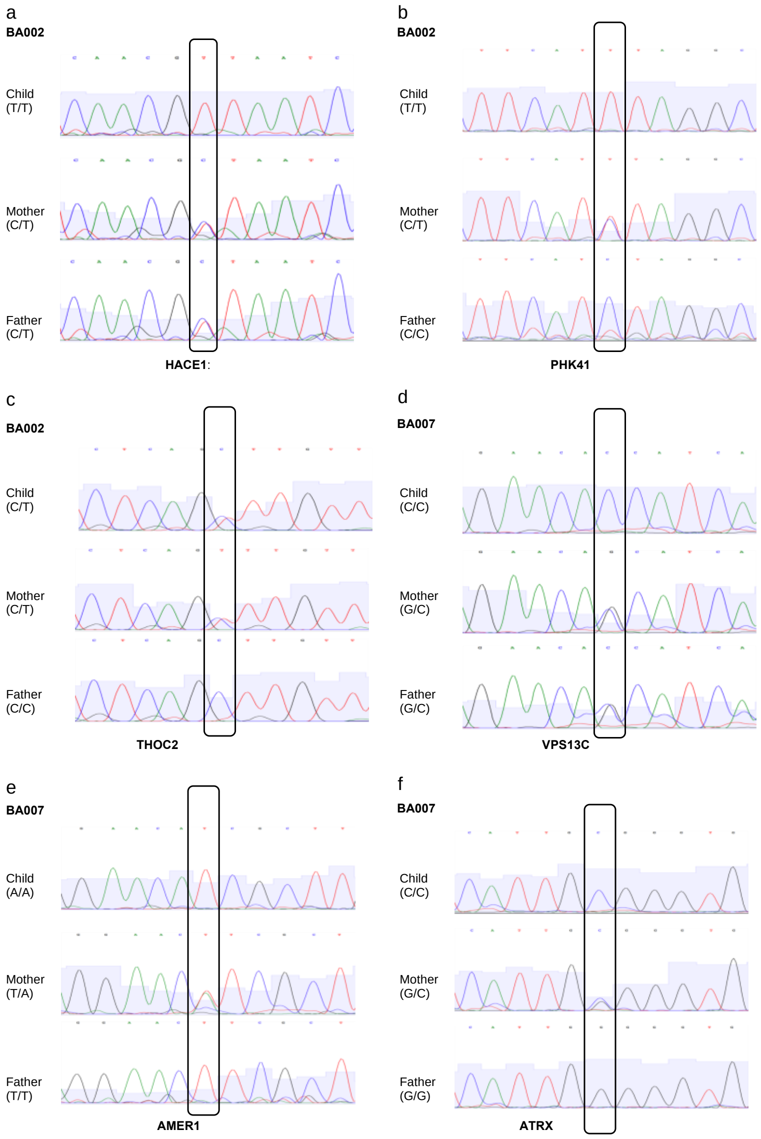
a
BA002
Child
(T/T)
Mother
(C/T)
Father
(C/T)
HACE1:
b
BA002
Child
(T/T)
Mother
(C/T)
Father
(C/C)
PHK41
c
BA002
Child
(C/T)
Mother
(C/T)
Father
(C/C)
THOC2
d
BA007
Child
(C/C)
Mother
(G/C)
Father
(G/C)
VPS13C
e
BA007
Child
(A/A)
Mother
(T/A)
Father
(T/T)
AMER1
f
BA007
Child
(C/C)
Mother
(G/C)
Father
(G/G)
ATRX

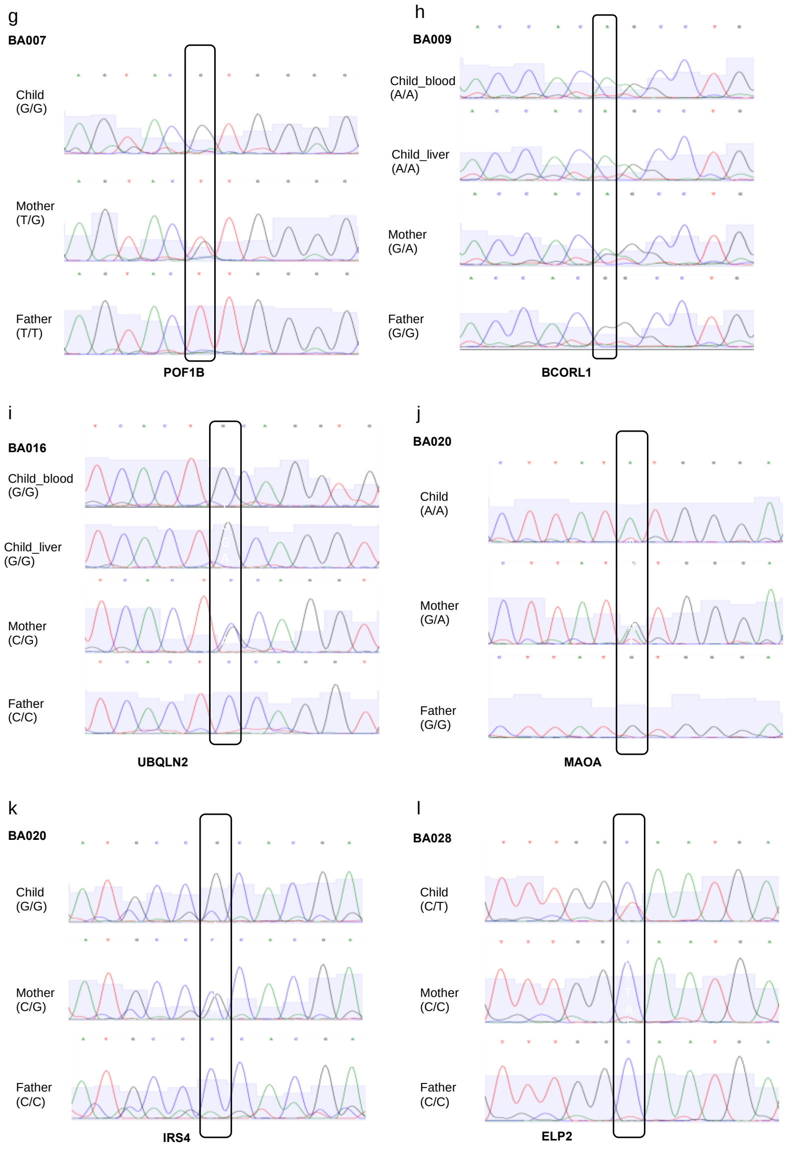
g
BA007
Child
(G/G)
Mother
(T/G)
Father
(T/T)
POF1B
h
BA009
Child_blood
(A/A)
Child_liver
(A/A)
Mother
(G/A)
Father
(G/G)
BCORL1
i
BA016
Child_blood
(G/G)
Child_liver
(G/G)
Mother
(C/G)
Father
(C/C)
UBQLN2
j
BA020
Child
(A/A)
Mother
(G/A)
Father
(G/G)
MAOA
k
BA020
Child
(G/G)
Mother
(C/G)
Father
(C/C)
IRS4
l
BA028
Child
(C/T)
Mother
(C/C)
Father
(C/C)
ELP2

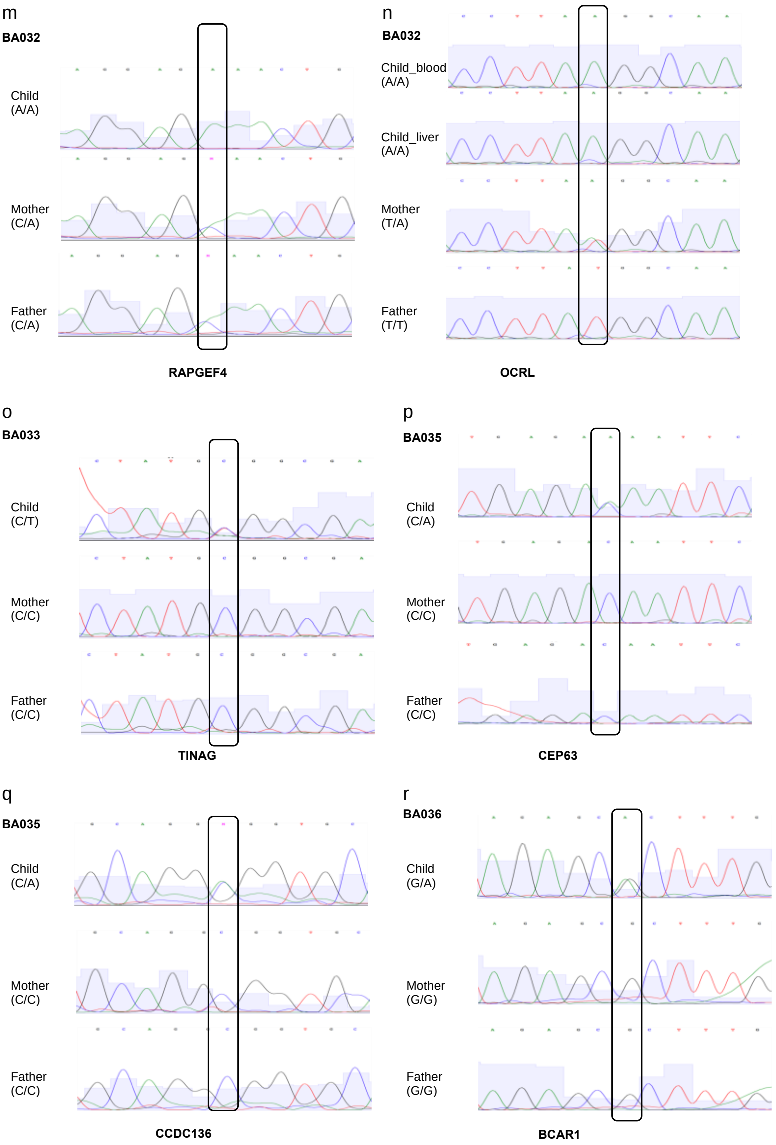
m
BA032
Child
(A/A)
Mother
(C/A)
Father
(C/A)
RAPGEF4
n
BA032
Child_blood
(A/A)
Child_liver
(A/A)
Mother
(T/A)
Father
(T/T)
OCRL
o
BA033
Child
(C/T)
Mother
(C/C)
Father
(C/C)
TINAG
p
BA035
Child
(C/A)
Mother
(C/C)
Father
(C/C)
CEP63
q
BA035
Child
(C/A)
Mother
(C/C)
Father
(C/C)
CCDC136
r
BA036
Child
(G/A)
Mother
(G/G)
Father
(G/G)
BCAR1

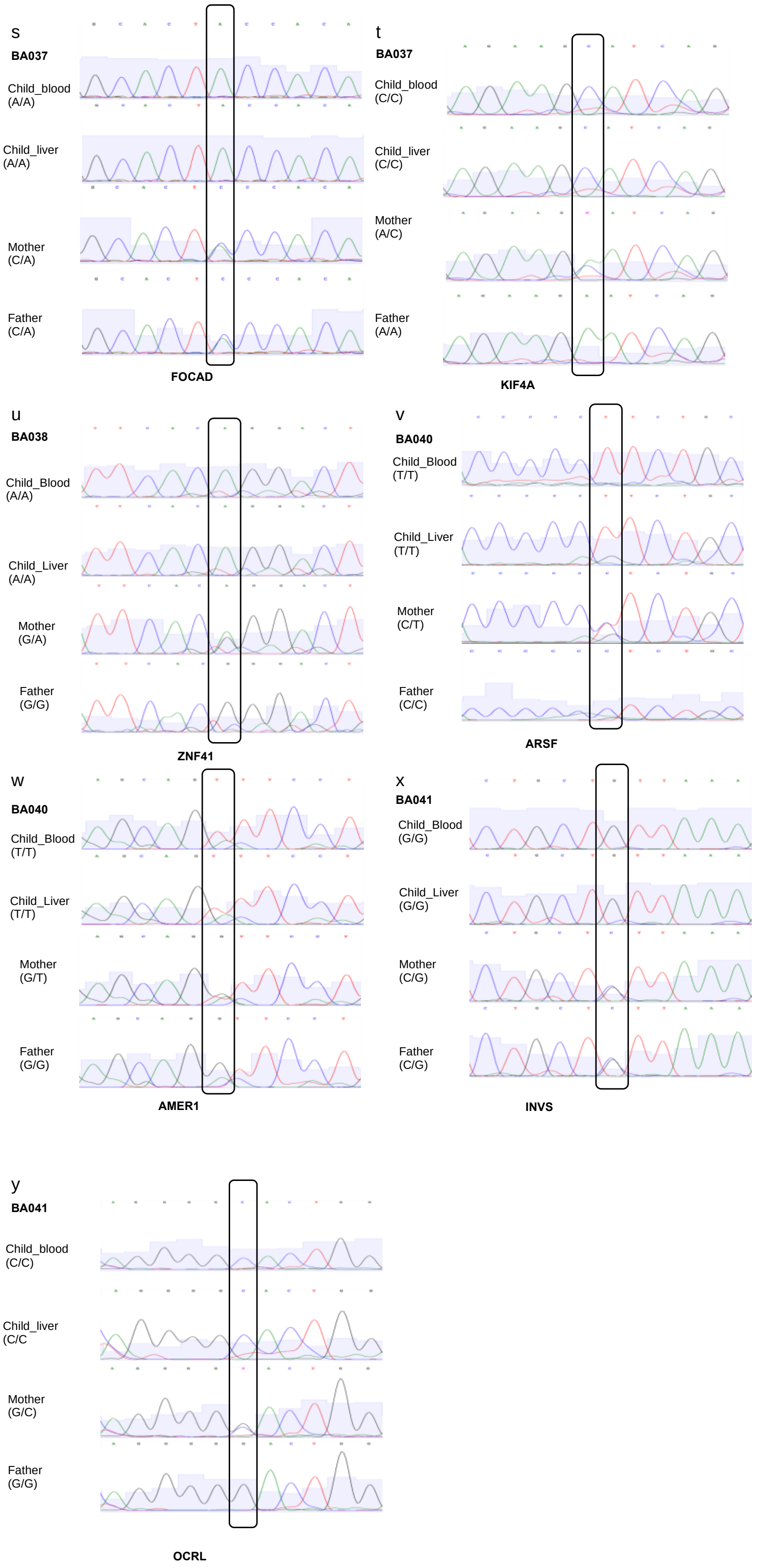

Fig S1. Sanger validation for indentified variants. The validations were performed on identified variants for child parent (on the left), and the genotype of each individual/sample is presented in parenthesis; the black square indicates the predisposition; the indentified gene is shown at the end of each subfigure.

Gene	Residue	Structure	Protein image	Amino acid properties
HACE1	p.Ala651Thr	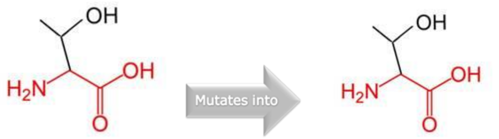	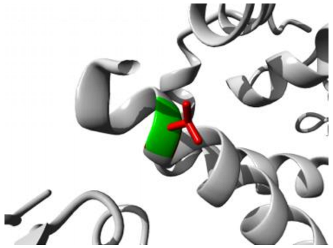	• The WT residue is more hydrophobic than the mutant residue<br>• The mutant residue is bigger than the WT residue. The mutation can disturb this domain and abolish its function.<br>• The mutated residue is located in a domain that is important for the main activity of the protein. Mutation of the residue might disturb this function.
PHKA1	p.Asp160Asn	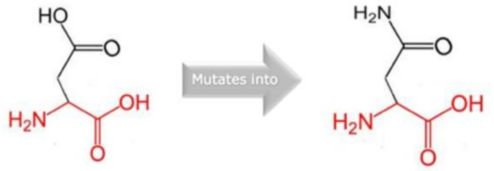	N/A	• The WT residue charge was NEGATIVE, the mutant residue charge is NEUTRAL.<br>• The mutant residue is located near a highly conserved position.<br>• The mutated residue is located in a domain that is important for binding of other molecules. Mutation of the residue might disturb this function.
THOC2	p.Ala421Thr	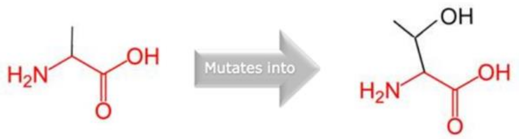	N/A	• The mutant residue is bigger than the WT residue.<br>• The WT residue is more hydrophobic than the mutant residue.<br>• The mutation converts the WT residue in a residue that does not prefer α-helices as secondary structure.
XIAP	p.Ala321Gly	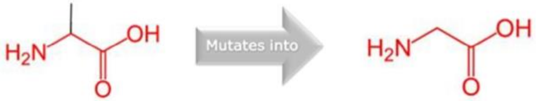	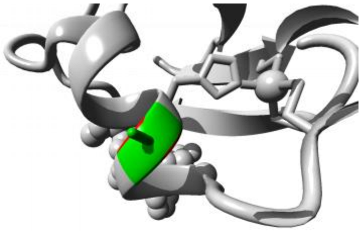	• The mutant residue is smaller than the WT residue.<br>• The mutation will cause an empty space in the core of the protein.<br>• The mutation will cause loss of hydrophobic interactions in the core of the protein.

VPS13C	p.Ala2043Gly	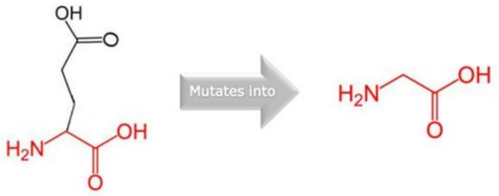	N/A	• The charge of the WT residue will be lost, this can cause loss of interactions with other molecules or residues.<br>• The mutant residue is smaller. This might lead to loss of interactions.<br>• The mutation introduces a more hydrophobic residue at this position. This can result in loss of hydrogen bonds and/or disturb correct folding.
AMER1	p.Ser359Cys	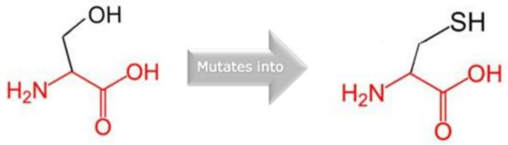	N/A	• The mutant residue is more hydrophobic than the WT residue.<br>• Based on this conservation information this mutation is probably damaging to the protein.<br>• The mutant residue is located near a highly conserved position.
ATRX	p.Pro2478Ala	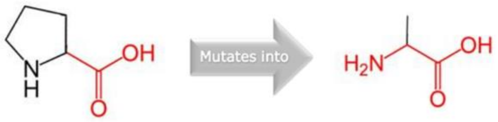	N/A	• The mutant residue is smaller. This might lead to loss of interactions.<br>• The mutation can disturb this special conformation.
POF1B	p.Ser109Arg	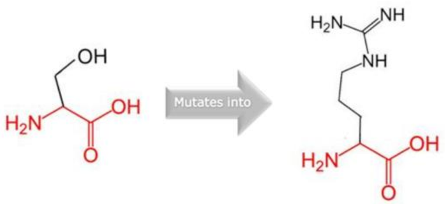	N/A	• The mutation introduces a charge. This can cause repulsion of ligands or other residues with the same charge.<br>• The mutant residue is bigger. This might lead to bumps.<br>• The hydrophobicity of the WT and mutant residue differs. Hydrophobic interactions, either in the core of the protein or on the surface, will be lost.
BCORL1	p.Arg890Gln	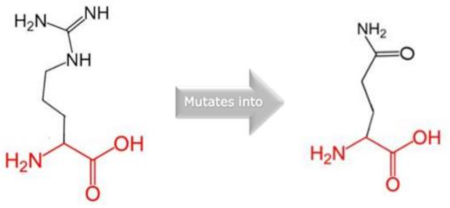	N/A	• There is a difference in charge between the WT and mutant amino acid.<br>• The mutant residue is smaller. This might lead to loss of interactions.<br>• The mutant residue is located near a highly conserved position.

INVS	p.Arg396*	N/A	N/A	N/A
BCOR	p.Pro483Leu	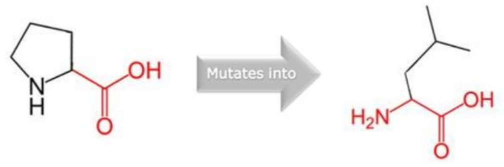	N/A	• The mutation can disturb this special conformation.<br>• The mutant residue is bigger, this might lead to bumps.
UBQLN2	p.Pro478Ala	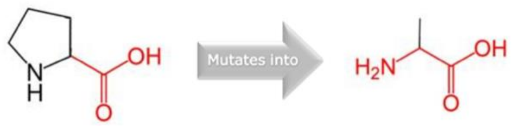	N/A	• The mutant residue is smaller. This might lead to loss of interactions.<br>• The mutation changes a proline with such a function into another residue, thereby disturbing the local structure.
MAOA	p.V70M	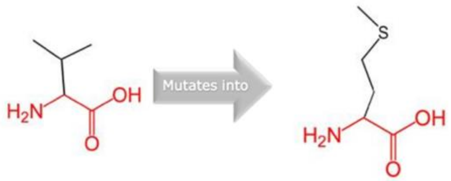	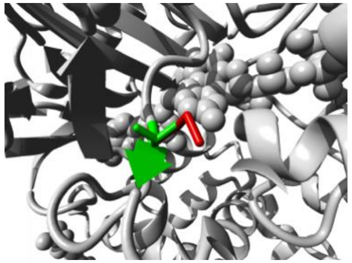	• The mutant residue is bigger than the WT residue. The WT residue was buried in the core of the protein.<br>• The mutant residue is located near a highly conserved position.<br>• The mutant residue prefers to be in another secondary structure, therefore the local conformation will be slightly destabilized.
IRS4	p.Trp945Cys	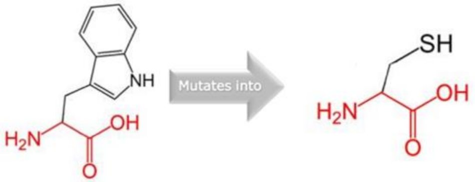	N/A	• The mutant residue is smaller. This might lead to loss of interactions.<br>• The mutant residue is located near a highly conserved position. This mutation is probably damaging to the protein.

ELP2	p.Ala445Val	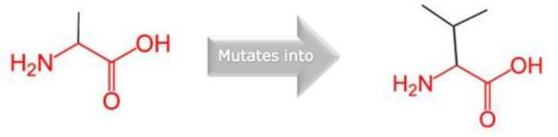	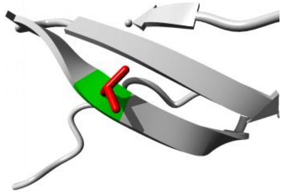	• The mutant residue is bigger. This might lead to bumps.<br>• The mutated residue is located in a domain that is important for binding of other molecules. Mutation of the residue might disturb this function.
RAPGEF4	p.Gln622Lys	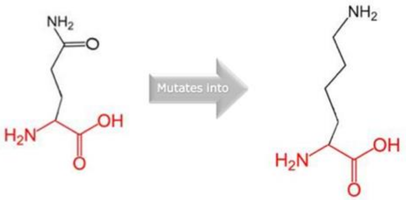	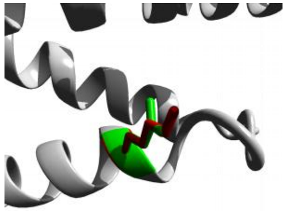	• The mutation introduces a charge. This can cause repulsion of ligands or other residues with the same charge.<br>• The mutant residue is bigger, this might lead to bumps
OCRL	p.Met876Lys	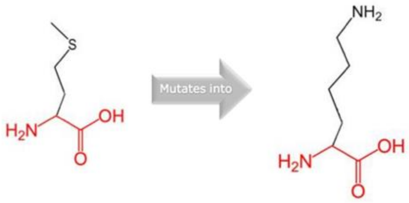	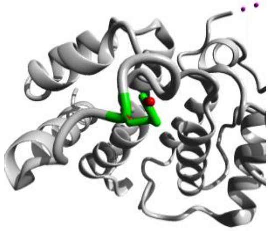	• The mutant residue is bigger than the WT residue.<br>• The residue is located on the surface of the protein, mutation of this residue can disturb interactions with other molecules or other parts of the protein.<br>• The mutation might cause loss of hydrophobic interactions with other molecules on the surface of the protein.
TINAG	p.Ala76Val	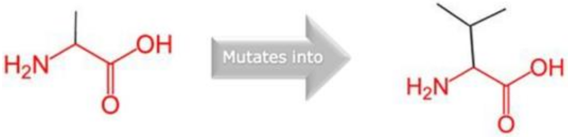	N/A	• The mutant residue is bigger. This might lead to bumps.<br>• The mutation introduces an amino acid with different properties, which can disturb this domain and abolish its function.

CEP63	p.Gln490Lys	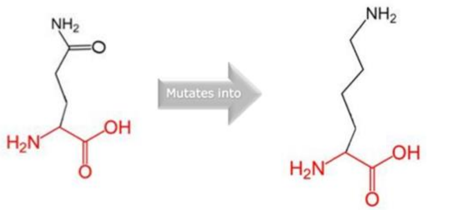	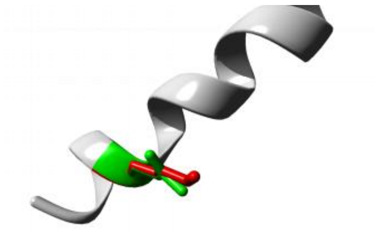	• The WT residue charge was NEUTRAL, the mutant residue charge is POSITIVE. The mutation introduces a charge. This can cause repulsion of ligands or other residues with the same charge.<br>• The mutant residue is bigger, this might lead to bumps.
CCDC136	p.Ala862Glu	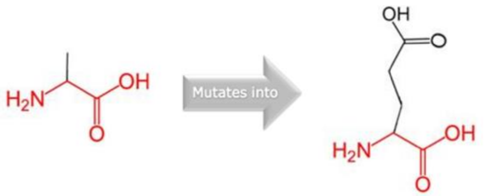	N/A	• The mutation introduces a charge. This can cause repulsion of ligands or other residues with the same charge.<br>• The mutant residue is located near a highly conserved position.<br>• The mutant residue is bigger. This might lead to bumps.<br>• Hydrophobic interactions, either in the core of the protein or on the surface, will be lost.
BCAR1	p.Ala10Val	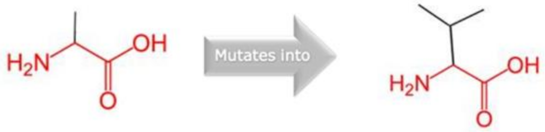	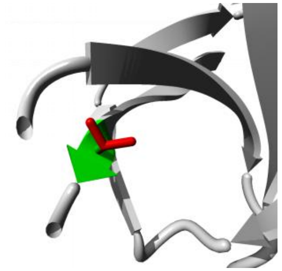	• The WT residue was buried in the core of the protein. The mutant residue is bigger and probably will not fit.<br>• The mutated residue is located in a domain that is important for binding of other molecules. The mutated residue is in contact with residues in another domain. It is possible that the mutation disturbs these contacts.
FOCAD	p.Pro1269Thr	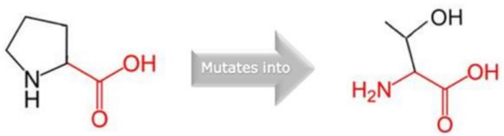	N/A	• Hydrophobic interactions, either in the core of the protein or on the surface, will be lost.<br>• The mutant residue is located near a highly conserved position.<br>• The mutation changes a proline with such a function into another residue, thereby disturbing the local structure.

KIF4A	p.Asn392His	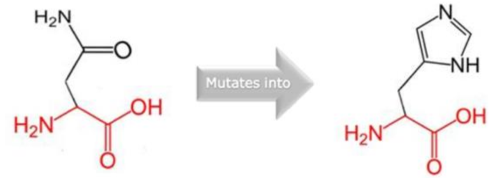	N/A	• The mutated residue is located in a domain that is important for the main activity of the protein. Mutation of the residue might disturb this function.<br>• The mutant residue is bigger, this might lead to bumps.
ZNF41	p.Arg299Cys	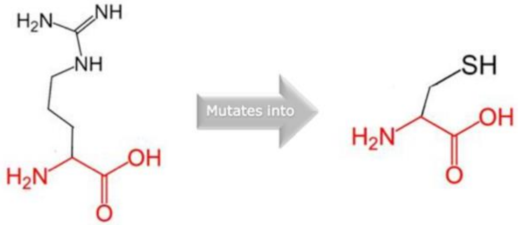	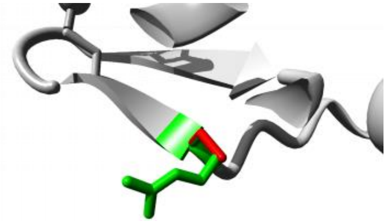	• The WT residue charge was POSITIVE, the mutant residue charge is NEUTRAL. The charge of the WT residue will be lost, this can cause loss of interactions with other molecules or residues.<br>• The mutation introduces a more hydrophobic residue at this position. This can result in loss of hydrogen bonds and/or disturb correct folding.<br>• The mutant residue is smaller, this might lead to loss of interactions.
ARSF	p.Pro504Leu	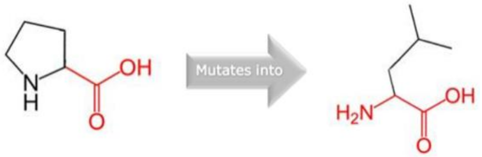	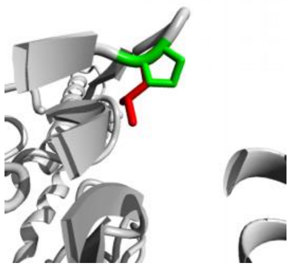	• The mutant residue is bigger. This might lead to bumps.<br>• The mutant residue is located near a highly conserved position. The mutated residue is located in a domain that is important for the main activity of the protein. Mutation of the residue might disturb this function.
AMER1	p.Thr708Asn	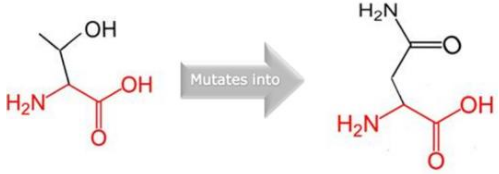	N/A	• The mutant residue is located near a highly conserved position.<br>• The mutant residue prefers to be in another secondary structure, therefore the local conformation will be slightly destabilized.<br>• The mutant residue is bigger. This might lead to bumps.<br>• Hydrophobic interactions, either in the core of the protein or on the surface, will be lost.

INVS	p.Leu40Val	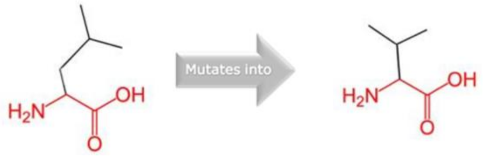	N/A	• The mutant residue is smaller than the WT residue. This might lead to loss of interactions.
OCRL	p.Asp89His	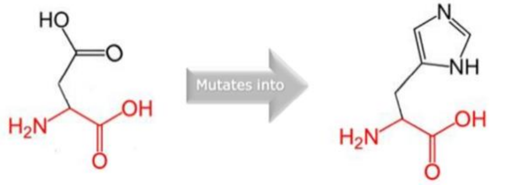	N/A	• The mutant residue is bigger. This might lead to bumps.<br>• The WT residue charge was NEGATIVE, the mutant residue charge is NEUTRAL. The charge of the WT residue will be lost, this can cause loss of interactions with other molecules or residues.

Fig S2. Predictions of structural changes for the identified variants (N/A: not available; WT: wild-type).
